# Using stable oxygen isotope dual‐inlet isotope‐ratio mass spectrometry to elucidate uranium transport and mixed ^230^Th/U calcite formation ages at the seminal Devils Hole, Nevada, natural laboratory

**DOI:** 10.1002/rcm.9926

**Published:** 2024-11-23

**Authors:** Tyler B. Coplen, Robert R. Seal, Lauren T. Reid, James A. Jordan, Adam C. Mumford

**Affiliations:** ^1^ U.S. Geological Survey Reston Virginia USA; ^2^ U.S. Geological Survey Catonsville Maryland USA

## Abstract

**Rationale:**

Vein calcite in Devils Hole has been precipitating continuously in oxygen‐isotope equilibrium at a constant temperature for over 500 000 years, providing an unmatched *δ* ^18^O paleoclimate time series. A substantial issue is that coeval calcite (based on matching *δ* ^18^O values) has uranium‐series ages differing by 12 000 years.

**Methods:**

An unparalleled high‐accuracy *δ* ^18^O chronology series from continuously submerged calcite was used to correct the published uranium‐series ages of non‐continuously formed calcite in two cores, cyclically exposed by water‐table decline during glacial–interglacial transitions. This method relies on the premise that the *δ* ^18^O values of coevally precipitated calcite are identical, allowing matching calcite *δ* ^18^O values to establish formation ages.

**Results:**

Exposed calcite can have apparent ages that are 12 000 years too young due to unrecognized uranium mobility and resulting mixed ages identified in over 50 mixed uranium‐series ages from previous studies. Secondary uranium in fluids, sourced from the formation or dissolution of porous carbonate deposits (folia) with high uranium‐238 (^238^U) concentrations, has migrated up to 10 mm into vein calcite.

**Conclusions:**

The continuously submerged Devils Hole *δ* ^18^O chronology is not explained by orbital forcing. Rather, this chronology represents a regional climate record in the southern Great Basin of sea‐surface‐temperature (SST) variations off California, variations that preceded the last and penultimate deglaciations by 5000 to approximately 10 000 years. Temporal discrepancies between the continuously submerged Devils Hole chronology and other regional *δ* ^18^O records (e.g., the Leviathan chronology) can be explained by unrecognized cryptic, pernicious uranium mobility, leading to model estimations that may be thousands of years younger than actual ages. Consequently, paleo‐moisture availability, water‐table, and groundwater recharge models based on these mixed uranium‐series ages are too young by as much as 12 000 years. The potential for post‐formation uranium addition in subaerial cores and speleothems underscores the need for caution in uranium‐series dating, highlighting *δ* ^18^O time‐series comparisons as a method for identifying mixed ages.

## 1 INTRODUCTION

**FIGURE 1:**
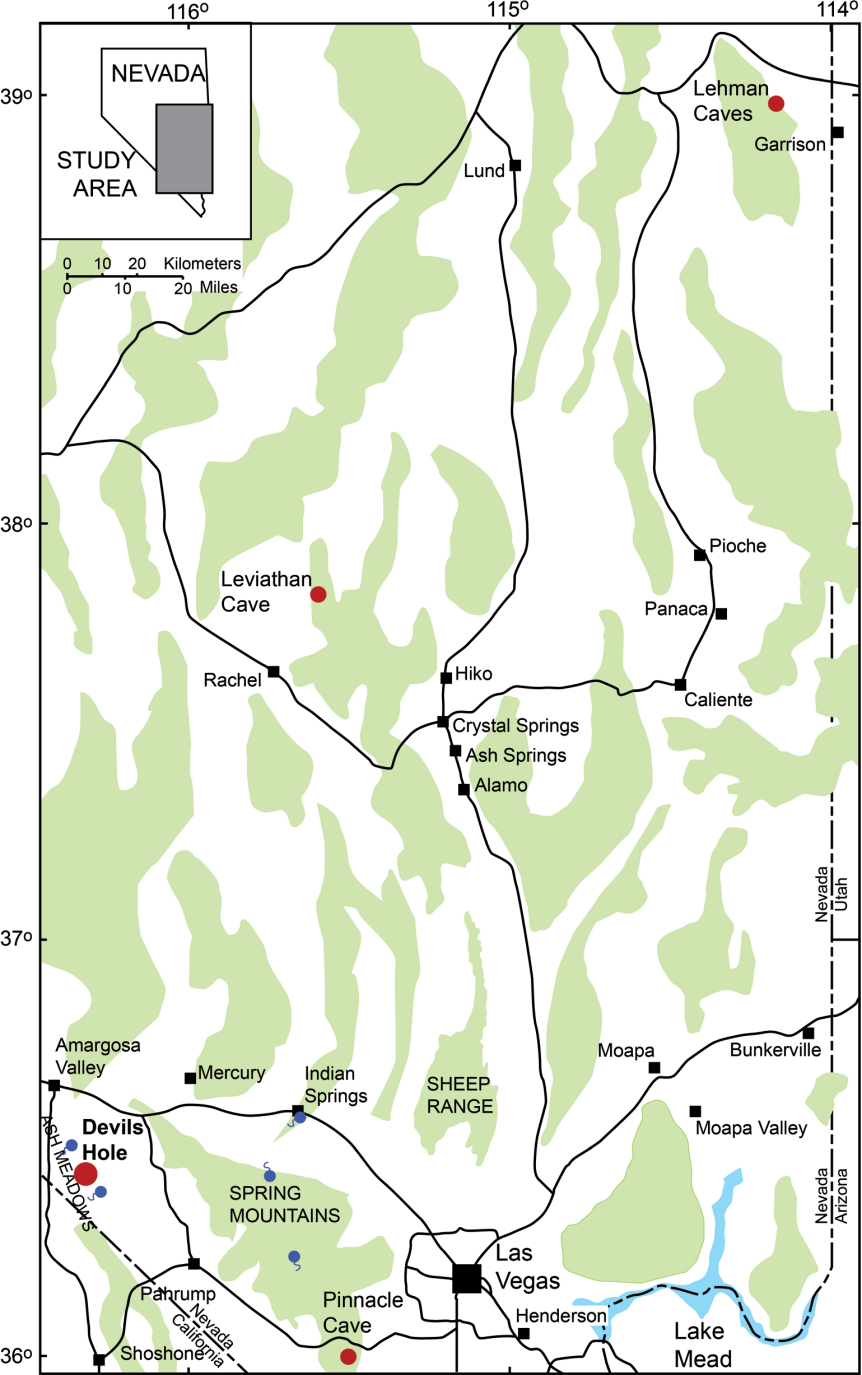
Index map of the southern Great Basin showing the locations of Devils Hole, significant springs, and the major mountains. Devils Hole Cave 2 is located about 200 m north of Devils Hole and lies within the filled red‐brown circle labeled “Devils Hole.” Regions in green indicate elevations higher than approximately 1500 m. Significant springs are shown as tadpoles. The primary source of groundwater flowing through Devils Hole, and discharging from the major springs within the oasis, is precipitation on the Spring Mountains and Sheep Range.^54^, ^55^, ^66^ The southern Great Basin composite dripstone record, the Leviathan chronology,^58^, ^59^, ^60^ was developed from speleothems from three Nevada caves: Lehman Caves, Leviathan Cave, and Pinnacle Cave. [Color figure can be viewed at wileyonlinelibrary.com]

**FIGURE 2:**
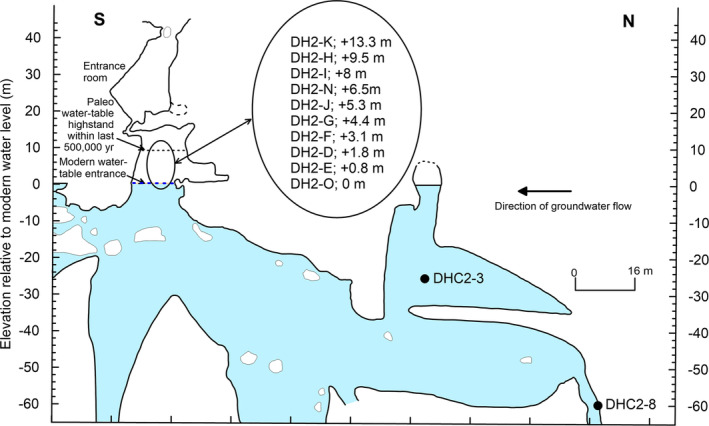
Cross‐sectional sketch of Devils Hole Cave 2. Modified from fig. 3 of Coplen et al.^12^ Cores DHC2‐3 (−25 m) and DHC2‐8 (−60 m) are from Winograd et al.^7^ Cores DH2‐O (0 m), DH2‐E (+0.8 m), DH2‐D (+1.8 m), DH2‐F (+3.1 m), DH2‐G (+4.4 m), DH2‐J (+5.3 m), DH2‐N (+6.5 m), DH2‐I (+8 m), and DH2‐H (+9.5 m) are from Moseley et al.^16^, ^17^ and Wendt et al.^18^, ^19^ Core DH2‐K (+13.3 m) is from Steidle et al.^20^, ^21^ Measured distances above the modern water table of several of the cores mentioned were corrected by Wendt et al.^19^ from that originally reported by Moseley et al.^16^, ^17^ The modern water‐table entrance to Devils Hole Cave 2 is shown as a blue dashed line. [Color figure can be viewed at wileyonlinelibrary.com]

For more than a half million years, dense vein calcite has been precipitating in Devils Hole, located about 115 km northwest of Las Vegas, Nevada. This process also occurs in Devils Hole Cave 2, which is hydrologically connected and situated about 200 m north of the original Devils Hole cave (Figures 1 and 2).^1^ Calcite has been precipitating in this natural laboratory in oxygen isotopic equilibrium,^2^, ^3^ at a constant temperature (±1°C)^4^ to a depth of at least 140 m.^5^ This vein calcite, also called mammillary calcite because of its morphology, is suitable for high‐accuracy uranium‐series (thorium‐230 [^230^Th]/U) dating.^6^, ^7^ Continuously submerged calcite contains an unbroken record of the sequential variation of the *δ* ^18^O of water recharging this well‐mixed natural laboratory.^1^, ^6^, ^7^, ^8^, ^9^, ^10^, ^11^, ^12^ Based on publications through 2011,^6^, ^7^, ^8^, ^9^, ^10^, ^11^, ^13^ the *δ* ^18^O of coevally precipitated calcite throughout the 140 m of the explored depth of Devils Hole and Devils Hole Cave 2 was expected to be identical within analytical uncertainty. This is exemplified by the good agreement of the *δ* ^18^O time series of cores DHC2‐3 (−25 m) and DHC2‐8 (−60 m) as illustrated by fig. 4 of Coplen et al.^12^ Information on all cores discussed here is found in Table S1 of Supporting Information.^14^

**FIGURE 3:**
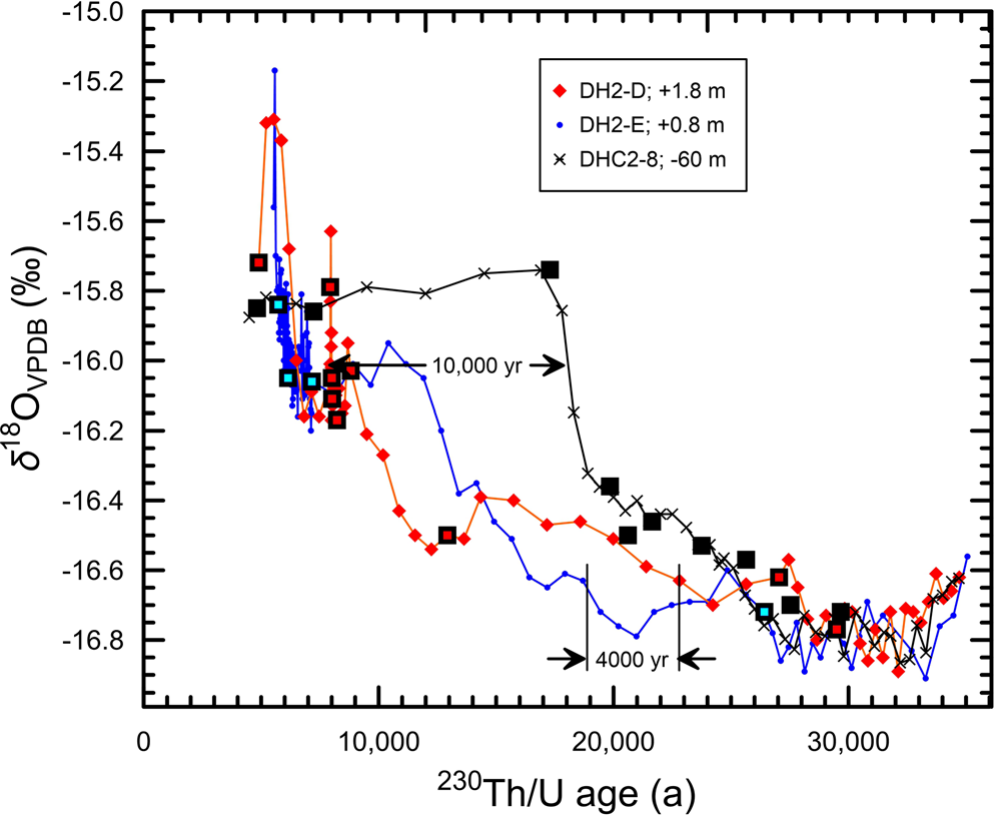
Detailed comparison of the *δ* ^18^O time series of deep and subaerial cores between 30 and 4.9 ka. Data for DH2‐D and DH2‐E are from Moseley et al.^16^ Data for DHC2‐8 are from Landwehr et al.^11^ and Coplen et al.^12^ Individual ^230^Th/U age measurements of samples from cores DH2‐E, DH2‐D, DHC2‐3, and DHC2‐8^11^, ^17^ are shown as open black squares, respectively, with cyan, red, and black centers. The 4000‐year difference in ages between cores DH2‐D and DH2‐E is determined from the DH2‐D sample at 153.20 mm having a *δ* ^18^O value of −16.63‰ and an age of 22 807 ± 1302 years (1‐*σ*)^16^ and the DH2‐E sample at 128.00 mm having a *δ* ^18^O value of −16.63‰ and an age of 18 690 ± 2908 years (1‐*σ*).^16^ To reconcile the DH2‐D and DH2‐E *δ* ^18^O time series with that of DHC2‐8, which is considered to be the “gold‐standard” *δ* ^18^O time series (see text), the DH2‐D and DH2‐E *δ* ^18^O time series must be moved to the right. [Color figure can be viewed at wileyonlinelibrary.com]

Since the most recent glacial, the water table dropped about 10 m,^15^ which exposed calcite in Devils Hole Cave 2. Nine cores were collected between 0 and 9.5 m above the modern water table in Devils Hole Cave 2 (Figure 2) by Moseley et al.^16^, ^17^ and Wendt et al.^18^, ^19^ Steidle et al.^20^, ^21^ collected four more cores between 13.3 and 19.5 m above the modern water table. A major geochemical conundrum appeared in 2016 when Moseley et al.^16^, ^17^ published the *δ* ^18^O values (Figure 3 and Figure S1), *δ* ^13^C values, and high‐accuracy ^230^Th/U ages of calcite from cores DH2‐E and DH2‐D (Figure 2) collected 0.8 and 1.8 m, respectively, above the modern water table at Devils Hole Cave 2. One would expect the *δ* ^18^O of coevally precipitated calcite in cores DH2‐D (+1.8 m), DH2‐E (+0.8 m), and DHC2‐8 (−60 m) to be identical within analytical uncertainty. However, at a *δ* ^18^O value of −16.02‰, DH2‐D is younger than DHC2‐8 by 10 000 years (Figure 3). Additionally, the Moseley et al.^16^, ^17^ *δ* ^18^O values from cores DH2‐D and DH2‐E are not in agreement. At a *δ* ^18^O value of −16.63‰, DH2‐E (+0.8 m) is younger than DH2‐D (+1.8 m) by approximately 4000 years (Figure 3). How can coevally precipitated calcite in this well‐mixed natural setting^22^ have ^230^Th/U ages that differ by as much as 10 000 years as illustrated in Figure 3? The aim of this study is to identify the cause of this discrepancy by comparing the high‐accuracy stable oxygen isotope time series of continuously precipitated, continuously submerged calcite with the time series of calcite collected above the modern water table.

## 2 METHODS

### 2.1 Stable oxygen and carbon isotope‐ratio mass spectrometry measurements

At the Reston Stable Isotope Laboratory of the U.S. Geological Survey, calcite samples are analyzed by evolving carbon dioxide from 20‐mg calcite samples. This is done by reacting samples with 2 mL of 100% phosphoric acid at 25°C overnight in a constant‐temperature water bath.^23^, ^24^ The specific gravity of the acid should be 1.02, and the acid must be aged for at least 1 month before use.^23^ In‐house reference materials calibrated relative to NBS 18 carbonatite and NBS 19 calcite are interspersed among unknown samples for two‐point normalization.^25^ Water is separated from carbon dioxide cryogenically using a glass “penta” manifold, which enables five samples to be processed simultaneously. Carbon dioxide samples are then loaded onto a stainless steel 20‐port manifold connected to a DuPont 21‐491 mass spectrometer modified for isotope‐ratio analysis. Unlike most isotope‐ratio mass spectrometers, this instrument uses glass mercury pistons instead of stainless‐steel bellows. The large dynamic range of the mercury pistons allows the transfer of the majority of sample carbon dioxide into the sample piston without needing cryogenic transfer. Additionally, unlike most light element isotope‐ratio mass spectrometers, this mass spectrometer features a 90° electric sector for energy focusing.^26^ An ion beam monitor located between the electric sector and the magnetic sector enables the quantitative determination of contamination in the sample carbon dioxide to identify leakage of air or other contaminants, which can affect isotope‐delta values. This dual‐inlet isotope‐ratio mass spectrometer achieves *δ* ^18^O and *δ* ^13^C accuracies of 0.02‰ with replicate measurements of isotopically homogeneous calcite.^23^ All Devils Hole calcite samples were analyzed in duplicate, if not replicate. To be conservative, one‐sigma accuracies of the *δ* ^18^O and *δ* ^13^C measurements of Devils Hole calcite have been published as 0.07‰ and 0.05‰, respectively.^27^ Standard deviations of replicate measurements are estimated to be about half these values. New results^12^ were published in support of this journal article. To determine *δ* ^18^O values versus Vienna Peedee belemnite (VPDB), *δ* ^18^O_VPDB_ values herein are derived by solving the expression^25^

(1) $$\delta^{18}O_{\text{VPDB}}=0.97001\times \delta^{18}O_{\text{VSMOW}}-29.99‰$$

### 2.2 Bat guano and soil sampling and analysis

Samples of bat guano were collected^28^ in Devils Hole Cave 2 in areas located between the current water table and about 5 m above the water table (Figure S2). Soil samples were collected 9 m above the water table near the entrance to Devils Hole Cave 2. Measurements of the uranium concentration in bat guano and soil samples (Table S2) were performed by preparing samples by total dissolution using microwave digestion with concentrated ultrahigh‐purity nitric acid followed by dissolution to <2% acid with ASTM Type 1 ultrapure (18‐MΩ) water and analyzed by inductively coupled plasma mass spectrometry (ICP‐MS) using an Agilent 7900 ICP‐MS and appropriate reference solutions.

### 2.3 Reanalysis of geochemical data

Results of ^230^Th/U age‐measurement data of samples from cores DH2‐D (+1.8 m), DH2‐E (+0.8 m), DH2‐F (+3.1 m), DH2‐G (+4.4 m), DH2‐H (+9.5 m), DH2‐I (+8.0 m), DH2‐J (+5.3 m), DH2‐K (+13.3 m), DH2‐N (+6.5 m), and DH2‐O (0 m), collected by Moseley et al.,^16^, ^17^ Wendt et al.,^18^, ^19^ and Steidle et al.,^20^, ^21^ have been reassessed. The locations of these cores are shown in Figure 2. Measured distances above the modern water table of several of the subaerial cores discussed by Moseley et al.^16^, ^17^ were corrected by Wendt et al.^19^ To our knowledge, measurements by Moseley et al.,^16^, ^17^ Wendt et al.,^18^, ^19^ and Steidle et al.^20^, ^21^ of uranium‐238 (^238^U) mass fractions, thorium‐232 (^232^Th) mass fractions, *n*(^230^Th)/*n*(^232^Th) isotope ratios, *δ* ^234^U activity values, and continuous‐flow isotope‐ratio mass spectrometry (CF‐IRMS) *δ* ^18^O_VPDB_ values are either excellent or fully satisfactory.

## 3 RESULTS

### 3.1 Corrected formation ages DH2‐D and DH2‐E samples younger than 30 ka

One would expect that the *δ* ^18^O values of coevally precipitated calcite in cores DH2‐D (+1.8 m), DH2‐E (+0.8 m), and DHC2‐8 (−60 m) to be identical within analytical uncertainty because they have been precipitated in oxygen isotopic equilibrium in a natural setting under stable conditions. Figure 3 shows that the *δ* ^18^O time series of DH2‐D (+1.8 m) and DH2‐E (+0.8 m) are not in temporal agreement, and neither is in temporal agreement with that of DHC2‐8 (−60 m). Because DHC2‐8 (−60 m) was continuously precipitated deep in the system in oxygen isotopic equilibrium,^2^, ^3^ has been continuously submerged, and was collected 110 m up the hydraulic gradient away from any influence of organic or inorganic compounds that might have entered the system from the entrance of Devils Hole Cave 2 (Figure 2), we conclude that the *δ* ^18^O time series of core DHC2‐8 (−60 m) is superior for use as a *δ* ^18^O time‐series calibrator.

**FIGURE 4:**
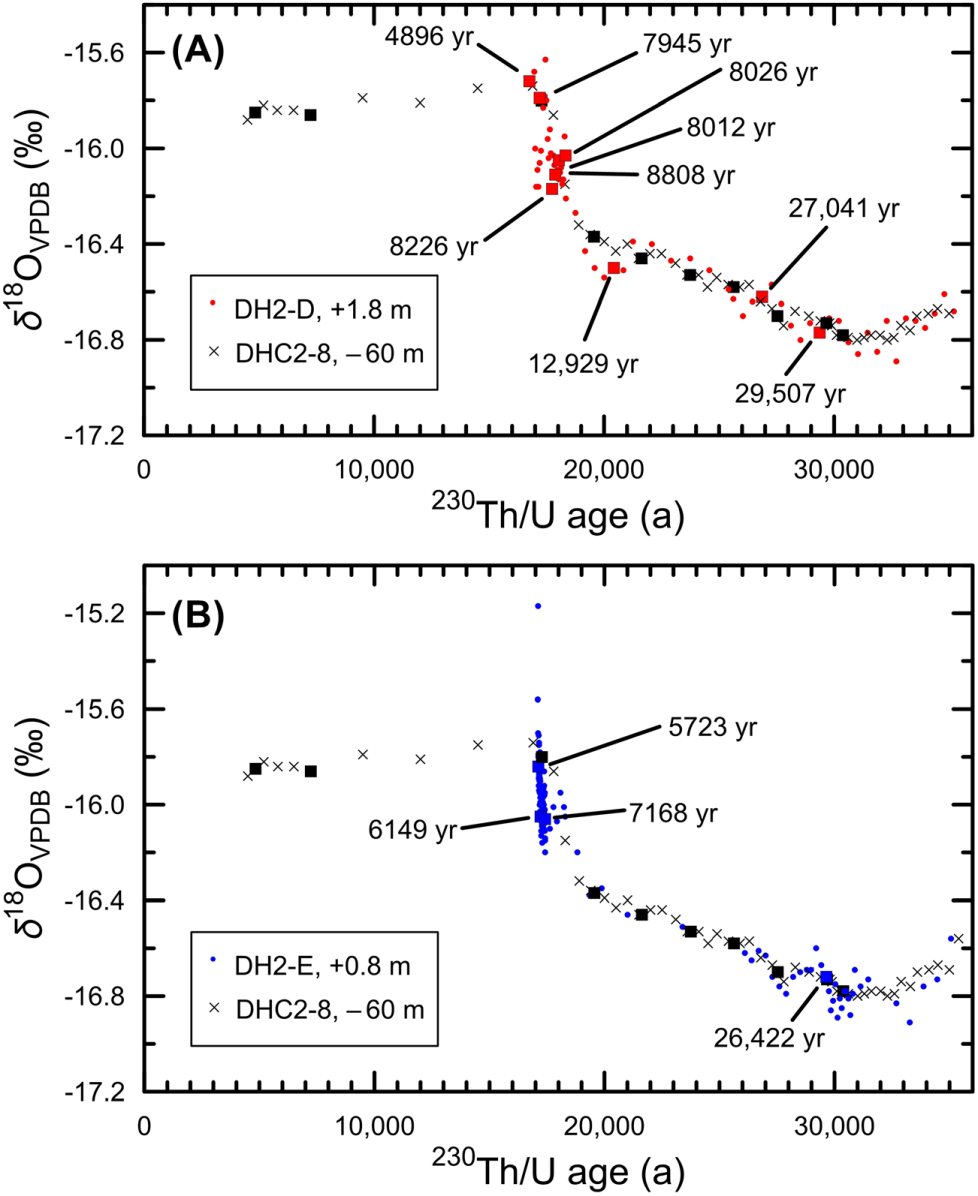
Estimated calcite formation ages of subaerial cores DH2‐D and DH2‐E between 35 and 4.5 ka based on the use of the *δ* ^18^O_VPDB_ time series of DHC2‐8 as a calibrator. The *δ* ^18^O_VPDB_ measurements of DHC2‐8 (−60 m) are from Landwehr et al.^11^ and Coplen et al.^12^ Ten ^230^Th/U age measurements of core DHC2‐8 (−60 m) are shown as solid black squares (Table S3).^7^, ^12^ (A) The *δ* ^18^O_VPDB_ measurements of DH2‐D (+1.8 m) samples are from Moseley et al.,^16^ and the nine ^230^Th/U ages^17^ are shown with open squares having red centers. (B) The *δ* ^18^O_VPDB_ measurements of DH2‐E (+0.8 m) samples are from Moseley et al.,^16^ and the four ^230^Th/U ages^17^ are shown with open squares having blue centers. [Color figure can be viewed at wileyonlinelibrary.com]

We estimated the corrected formation ages of calcite in cores DH2‐D (+1.8 m) and DH2‐E (+0.8 m) using the *δ* ^18^O time series of DHC2‐8 (−60 m)^12^ as a calibrator (Figure 4A,B). The *δ* ^18^O time series of DH2‐D (+1.8 m)^16^ and DHC2‐8 (−60 m) are in good agreement (Figure 4A). The major results from Figure 4A are the following: (1) Calcite ceased precipitating at about 17 ka in Devils Hole Cave 2, (2) the ^230^Th/U ages of the youngest seven ages of DH2‐D are too young by as much as 12 000 years,^17^ and (3) all the ^230^Th/U ages younger than about 21 ka are too young by thousands of years.

The *δ* ^18^O time series of DH2‐E (+0.8 m)^16^ and DHC2‐8 (−60 m) are in fair agreement (Figure 4B). Some of the *δ* ^18^O measurements of DH2‐E are more negative by about 0.1‰–0.2‰ than the dual‐inlet isotope‐ratio mass spectrometry (DI‐IRMS) measurements of DHC2‐8 (−60 m). The reason is unknown. The youngest *δ* ^18^O measurements of DH2‐E (+0.8 m) are as much as 0.5‰ more positive than the *δ* ^18^O measurements of DHC2‐8, and this may be a result of the development of “punk” carbonate.^29^ The major results from Figure 4B are the following: (1) Calcite ceased precipitating at about 17 ka in Devils Hole Cave 2, (2) the ^230^Th/U ages of the youngest three ages of DH2‐E are too young by as much as approximately 12 000 years, and (3) all the ^230^Th/U ages less than about 20 ka are too young by thousands of years.

### 3.2 Can the ^230^Th/U ages of DH2‐D and DH2‐E calcite samples younger than 21 ka be correct?

To explain temporal differences in the *δ* ^18^O time series of DH2‐D (+1.8 m) and DH2‐E (+0.8 m) shown in Figure 3 and Figure S1, Moseley et al.^16^ postulated that there was an increasing ^230^Th concentration down the water column, as has been observed in vertical profiles through the ocean water column.^30^, ^31^ Our evaluation indicates that there are several lines of evidence that suggest that this hypothesis cannot stand:

1. If ^230^Th concentrations were increasing down the water column at Termination II, the same would be expected during Termination I. Because the depth of DH2‐E is lower than that of DH2‐D, the ^230^Th/U ages of coevally formed DH2‐E (+0.8 m) samples would be expected to be greater than those of coeval DH2‐D (+1.8 m) samples. However, Warren D. Sharp (Berkeley Geochronology Center, personal communication) pointed out that the opposite situation occurs between 24 and 15 ka (Figure 3); the ^230^Th/U ages of DH2‐E (+0.8 m) samples are *younger* than those of coeval DH2‐D (+1.8 m) samples, when they should be older.
2. During two field campaigns in 2016 and 2017, seven water samples were collected between 0.2‐ and 90‐m depths.^32^, ^33^ Thorium was nearly non‐existent “at ~50 femtogram per gram” in all samples. The data of Dublyansky^32^, ^33^ in our view rule out the postulated increase of ^230^Th concentration down the water column.
3. Winograd^22^ documented that the active flow of geothermal water through the Devils Hole caves precludes the postulated increase in ^230^Th with depth. Additionally, the effects of regularly occurring earthquakes^34^, ^35^, ^36^, ^37^, ^38^, ^39^ and Earth tides preclude maintenance of a monotonically increasing gradient of ^230^Th concentration with depth, which Moseley et al.^16^ hypothesized and displayed in their fig. 4.
4. The Moseley et al.^17^, ^40^ 5‐year travel time between Devils Hole Cave 2 and the original Devils Hole in support of a ^230^Th gradient neglects to take into account the restricted cross‐sectional area of the aquifer in the vicinity of the major fault damming the aquifer and the dominant northeast (NE)–southwest (SW) direction of fracture permeability. The travel time is likely an order of magnitude quicker, precluding maintenance of the hypothesized ^230^Th gradient.

We are unable to find *any* evidence to support an increasing ^230^Th concentration down the water column. We conclude, as Winograd^22^ concluded, that groundwater in the Devils Hole caves is well mixed and no ^230^Th concentration gradient down the water column exists.

Because an increasing ^230^Th concentration down the water column is not supported, we must search elsewhere for an explanation for the differences among the *δ* ^18^O time series of the two subaerial cores and the deep cores. In his seminal review of uranium‐series dating, Schwarcz^41^ noted that a primary concern includes open‐system processes that would enable migration of uranium. For example, transport of uranium into a carbonate substantially after formation will yield a mixed‐age sample having an age younger than the formation age of the carbonate, a process termed post‐formation uranium addition. Our study describes the distribution of ^238^U in different types of Devils Hole carbonates that are relevant to accurate paleoclimate and geochemical modeling, and we evaluate the impact upon their measured ^230^Th/U ages.

### 3.3 Variability in the ^238^U concentrations of carbonates

**FIGURE 5:**
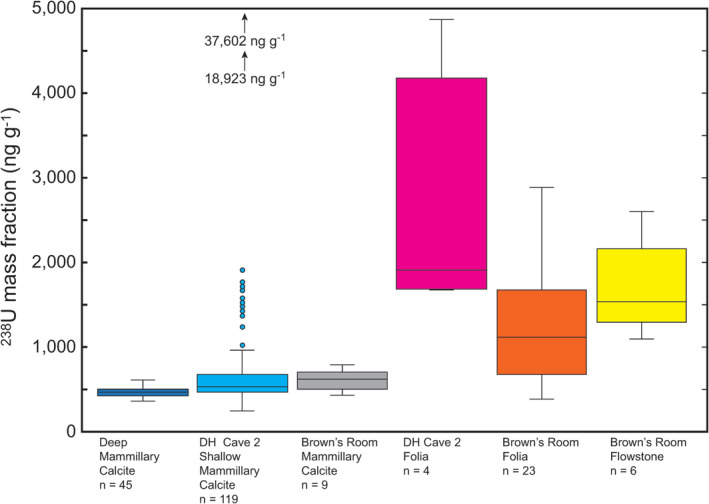
Box‐and‐whisker plots showing the ranges of ^238^U concentration in calcite, folia, and flowstone at Devils Hole. Data for Devils Hole and Devils Hole Cave 2 deep calcite are from DH‐2 (−21 m)^8^, ^17^ and from DHC2‐3 (−25 m) and DHC2‐8 (−60 m).^12^ Data for Devils Hole Cave 2 shallow calcite are from DH2‐D (+1.8 m), DH2‐E (+0.8 m), DH2‐F (+3.1 m), DH2‐G (+4.4 m), DH2‐H (+9.5 m), DH2‐I (+8.0 m), DH2‐J (+5.3 m), DH2‐N (+6.5 m), and DH2‐O (0 m) (see Figure 2) having ages younger than 182 ka.^17^, ^19^ The circles with blue centers depict samples having ^238^U mass fractions between 1000 and 1907 ng g^−1^. Devils Hole Cave 2 folia are from Moseley et al.^17^ Data for all Brown's Room samples in Devils Hole are from Szabo et al.,^15^ which were collected between 0 and 9 m above the modern water table. The uranium concentrations of Winograd et al.^8^ have been converted to ^238^U concentrations.^67^ [Color figure can be viewed at wileyonlinelibrary.com]

**FIGURE 6:**
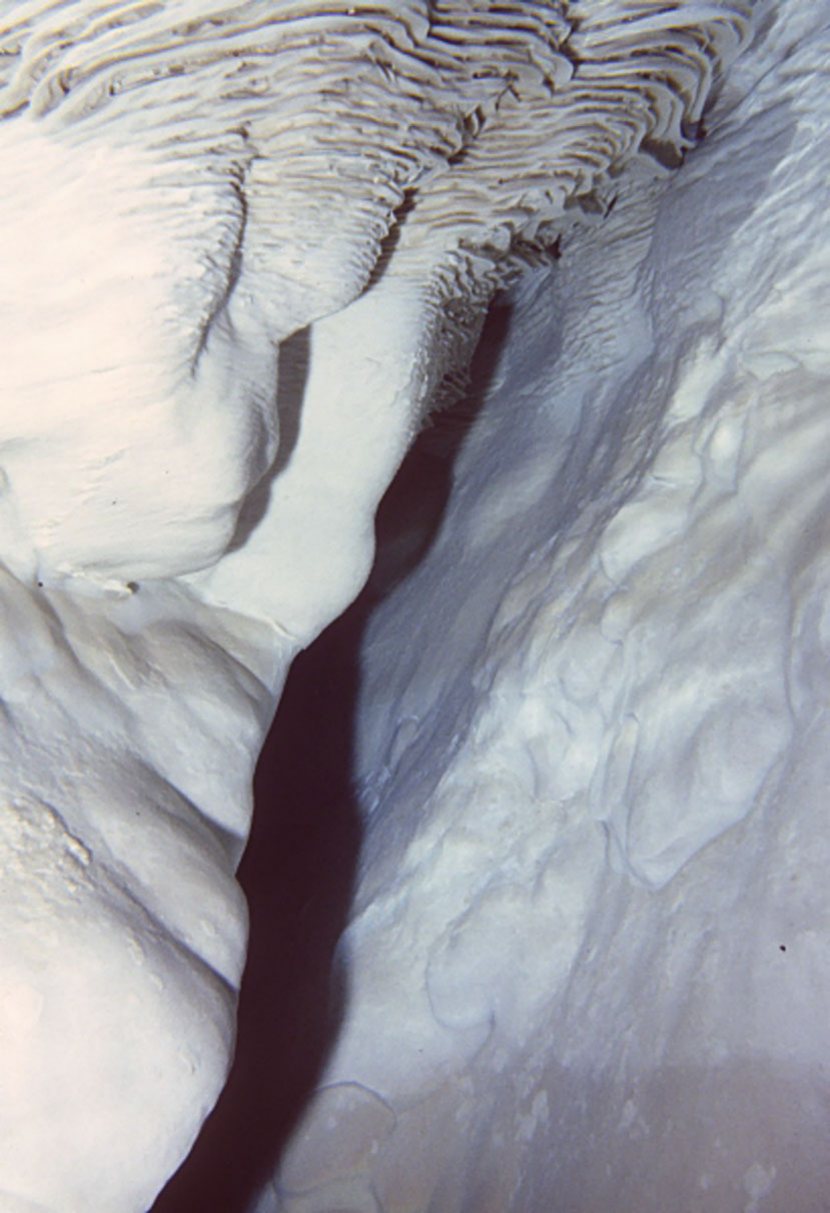
Vein calcite and folia. Photo by R. J. Hoffman (U.S. Geological Survey) taken in Brown's Room, a subaerial room in Devils Hole.^42^ Vein calcite (also termed mammillary calcite) coating shown in the lower three fourths of the photo. Folia (with ripples) are shown in the upper fourth of the photo. These folia extend about 1 m below the water table, indicating that the water table once stood a meter lower than the modern water‐level stand. The photograph was taken approximately 1 m below the water surface in the southwest corner of the pool in Brown's Room. The location of Brown's Room in Devils Hole is shown in fig. 1 of Coplen et al.^12^ The vertical distance in the photograph spans about 2 m. [Color figure can be viewed at wileyonlinelibrary.com]

Distinct differences are apparent in the ^238^U concentrations of calcite, folia, and flowstone from Devils Hole (Figure 5), including differences based on whether folia‐free, hiatus‐free (continuously precipitated) deep calcite (collected at depths greater than 20 m below the modern water table) or shallow calcite having interbedded folia and hiatuses (collected at or above the modern water table) were sampled. Calcite, termed vein calcite to denote its mode of origin and mammillary calcite to denote its morphology, is the term used herein. Calcite has been able to grow continuously on near‐vertical walls of Devils Hole and Devils Hole Cave 2 to thicknesses greater than 40 cm^9^, ^42^ from upwelling geothermal groundwater (34°C) that is slightly supersaturated with respect to calcite.^43^ Calcite forms a massive, uniformly thick coating of very coarsely crystalline calcite in the open fault zone (Figure 6). Calcite is the only coating present from approximately 1 m below the modern water table to the present limit of SCUBA exploration of about 140 m below the modern water table.^5^ Folia, porous carbonate deposits that resemble bracket fungi in shape (Figure 6), form on vertical to overhung surfaces and accumulate within the 3–11‐cm intertidal range of the water table.^5^, ^15^, ^44^ A falling water table deposits folia over previously submerged calcite in the intertidal zone of the cave. Previously submerged calcite is interbedded with folia to an elevation of about 9.5 m above the modern water table,^19^ indicating that the water table at Devils Hole was at least 10 m higher in the past (Figure 2). Steidle et al.^21^ reported calcite samples collected from an elevation of 19.5 m having ages >700 ka.

The median mass fraction of ^238^U within shallow calcite from Devils Hole Cave 2 having ages younger than 182 ka^17^, ^19^ is 521 ng g^−1^ (*n* = 119). High values range up to 37 602.3 ± 50.8 ng g^−1^,^19^ about 80 times the uranium concentration within deep calcite (Figure 5). Not included are about 50 measurements from Moseley et al.^17^ and Wendt et al.^19^ having ages greater than 182 ka, the approximate age of the oldest DH‐2 sample analyzed by Moseley et al.^17^ Mean ^238^U concentrations and variabilities in the concentrations of subaerially formed calcites (folia and flowstone) are the highest. Flowstone is carbonate that precipitates from films of calcite‐supersaturated water flowing down the walls of Brown's Room (fig. 1 of Coplen et al.^12^), a subaerial room in Devils Hole.^5^, ^45^ Deep calcite has the lowest variability in ^238^U concentration and the lowest ^238^U concentration. Therefore, the ^238^U concentration throughout the Devils Hole caves is expected to be homogeneous; the ^238^U concentration of coevally formed calcite between 1‐ and 140‐m depths should be very similar.

Does the temporal disagreement among *δ* ^18^O time series of deep and subaerial cores occur because the subaerial cores contain both primary uranium (from its formation) and secondary uranium added substantially after formation, resulting in mixed‐^230^Th/U‐age samples? If post‐formation uranium addition occurred in subaerial calcite, one would expect the ^238^U concentrations of mixed‐^230^Th/U‐age calcite samples to be greater than those of calcite samples containing only primary uranium.

### 3.4 ^238^U concentration of primary uranium

**FIGURE 7:**
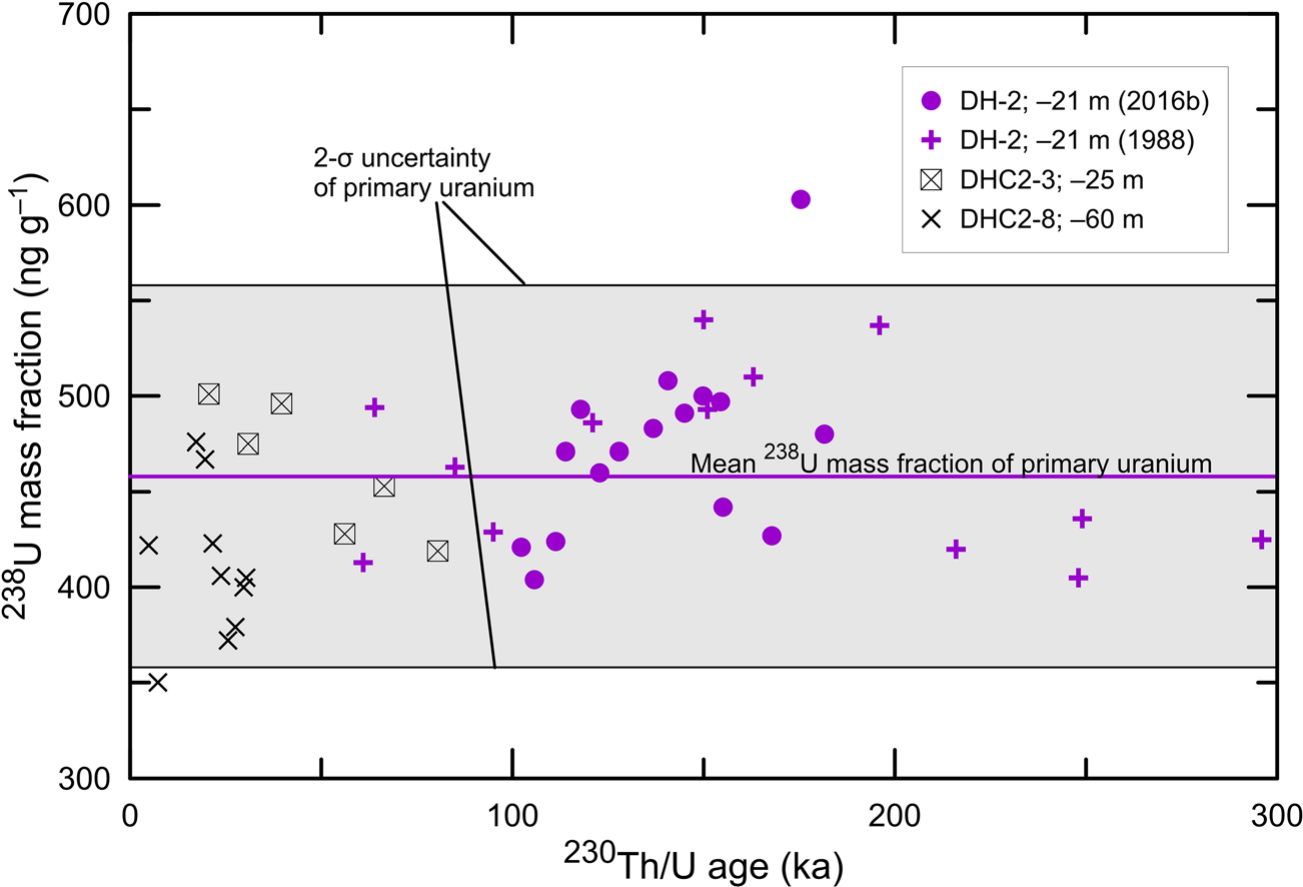
Crossplot of ^238^U mass fraction of calcite versus ^230^Th/U age as determined from deep cores DH‐2, DHC2‐3, and DHC2‐8. The mean ^238^U mass fraction of primary uranium within calcite is 456 ± 100 ng g^−1^ (2‐*σ* uncertainty, *n* = 45; see Table S3). Data for DH‐2 are from Winograd et al.^8^ and Moseley et al.^17^ Data for DHC2‐3 and DHC2‐8 are from Coplen et al.^12^ [Color figure can be viewed at wileyonlinelibrary.com]

A best estimate of primary uranium concentration was computed by evaluating data from calcite samples formed between 296 and 4.9 ka (Table S3). Samples having anomalously high uranium concentrations were excluded; for example, the ^238^U mass fraction of sample DH2‐O‐31,^19^ collected at the modern water table, is 37 602.3 ± 50.8 ng g^−1^. This ^238^U concentration is about 80 times that within samples from core DH‐2 (−21 m), which ranged from 404 to 603 ng g^−1^.^17^ To be conservative, calcite samples that were collected at or above the modern water table were excluded because their ^238^U concentrations may be higher than that of primary ^238^U within calcite. Included in our evaluation are the ^238^U concentrations of samples from deep cores DH‐2 (−21 m),^8^, ^17^ DHC2‐3 (−25 m), and DHC2‐8 (−60 m).^12^ The ^238^U concentration measurements of DH‐11 (−30 m) samples could not be located. The best estimate of the mean mass fraction of primary ^238^U within calcite (Figure 7) is 456 ± 100 ng g^−1^ (2‐*σ* uncertainty, *n* = 45). Therefore, the ^238^U mass fraction of primary uranium of 99% of calcite samples (3‐*σ* uncertainty) should lie between 306 and 606 ng g^−1^. Calcite samples having ^238^U mass fractions greater than 606 ng g^−1^ are identified herein as high‐^238^U calcites and are expected to contain secondary uranium, resulting in samples having mixed ^230^Th/U ages.

### 3.5 Samples with secondary uranium

The median of data used to calculate the best estimate of the ^238^U mass fraction of primary uranium within Devils Hole deep calcite samples is 460 ng g^−1^ (Figure 5). In contrast, the median mass fraction of ^238^U within Devils Hole Cave 2 shallow calcite, which includes data for DH2‐D (+1.8 m), DH2‐E (+0.8 m), DH2‐F (+3.1 m), DH2‐G (+4.4 m), DH2‐H (+9.5 m), DH2‐I (+8.0 m), DH2‐J (+5.3 m), DH2‐N (+6.5 m), and DH2‐O (0 m) (Figure 5), is 521 ng g^−1^ (*n* = 119).^17^, ^19^ However, two outlier values from Devils Hole Cave 2 range up to 37 602.3 ± 50.8 ng g^−1^ in Figure 5.^19^

**FIGURE 8:**
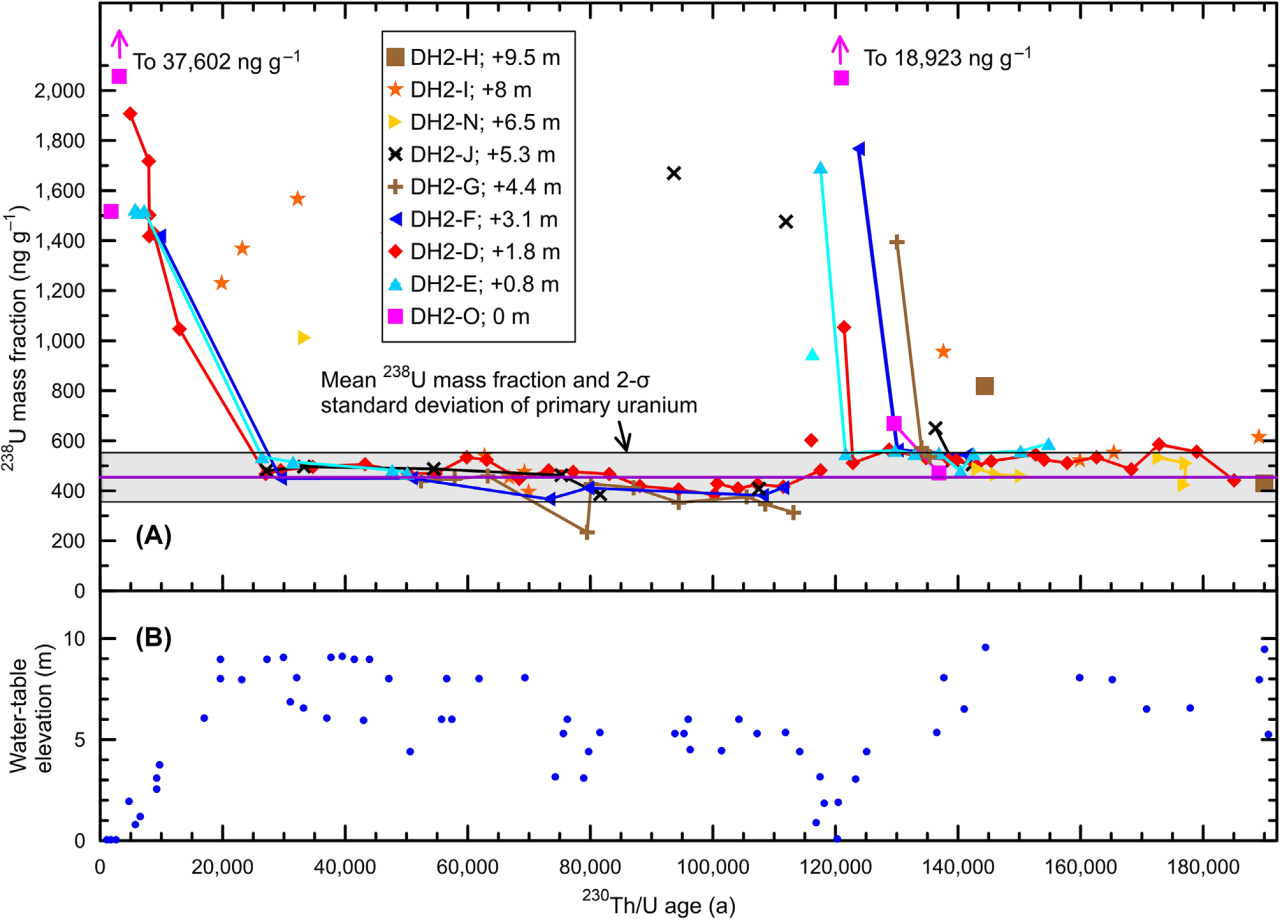
^238^U concentration and water‐table‐elevation time series. (A) Crossplot of ^238^U mass fraction of calcite versus ^230^Th/U age within cores collected at or above the modern water table in Devils Hole Cave 2.^17^, ^19^ (B) Paleowater‐table elevations relative to the modern water‐table level (data from Szabo et al.,^15^ Wendt et al.,^18^ and Jackson et al.^63^). [Color figure can be viewed at wileyonlinelibrary.com]

^230^Th/U‐age data from subaerial cores^17^, ^19^ indicate that there are 37 high‐^238^U (>606 ng g^−1^) calcite samples having ages less than 296 ka (Table S4) with ^238^U mass fractions as high as 37 602.3 ± 50.8 ng g^−1^. They are concentrated during the periods 145–115 ka and post‐25 ka, that is, near Terminations II and I and their interglacials (Figure 8A). These high‐^238^U‐concentration samples occur only in the nine subaerial cores (Figure 2), and their timing corresponds to the onset of Termination II (about 140–115 ka) and Termination I (post‐25 ka) and the concomitant lowering of the water table (Figure 8B). Additionally, there are nine samples having ages less than 296 ka (discussed in the next section) that have moderate ^238^U enrichment for a total of 46 samples having secondary uranium (Table S4). Table S5 lists an additional 14 samples older than 296 ka having estimated secondary uranium mass fractions as high as 1115 ng g^−1^.

## 4 DISCUSSION

### 4.1 Proposed infiltration of secondary uranium from folia into mammillary calcite and mixed‐age samples

All 37 high‐^238^U calcite samples and resulting mixed ages (Table S4) are from core samples that are adjacent to folia or contiguous with other high‐^238^U calcite samples.^17^, ^19^ The reason for this is that folia were precipitated over calcite in the intertidal zone.^5^ As the water table declined during the onset of interglacials, the calcite and adjacent folia were exposed above the water table (Figure 8A,B). The ^238^U mass fractions in these folia can be exceedingly high. Moseley et al.^17^ published ^238^U mass fractions of four folia samples in core DH2‐D of 1729 ± 2 (DH2‐D2‐2), 2084 ± 3 (DH2‐D2‐3), 4889 ± 4 (DH2‐D2‐4), and 1670 ± 2 ng g^−1^ (DH2‐D2‐5). The median mass fraction of ^238^U in these folia is 1907 ng g^−1^ (*n* = 4) (Figure 5), which is as much as a factor of 4 higher than that of primary calcite. Folia are porous to very porous,^5^, ^15^, ^17^ and one possibility is that fluids having high uranium concentrations filter into adjacent calcite when folia and calcite are at or above the water table either during intertidal fluctuations or during longer term drought conditions, promoting post‐formation uranium addition. A potential process producing these fluids having higher uranium concentrations is condensation corrosion,^29^ discussed in the next section. Diffusion of uranium, such as solid‐state diffusion, is unlikely. For enrichment of uranium in vein calcite, one needs only fluid traveling along oblique factures to access preferential binding sites. The fluids could be Devils Hole's recharge of surface water or water involved in some formation or dissolution of folia and enriched in uranium. Any of these would provide secondary uranium.

**FIGURE 9:**
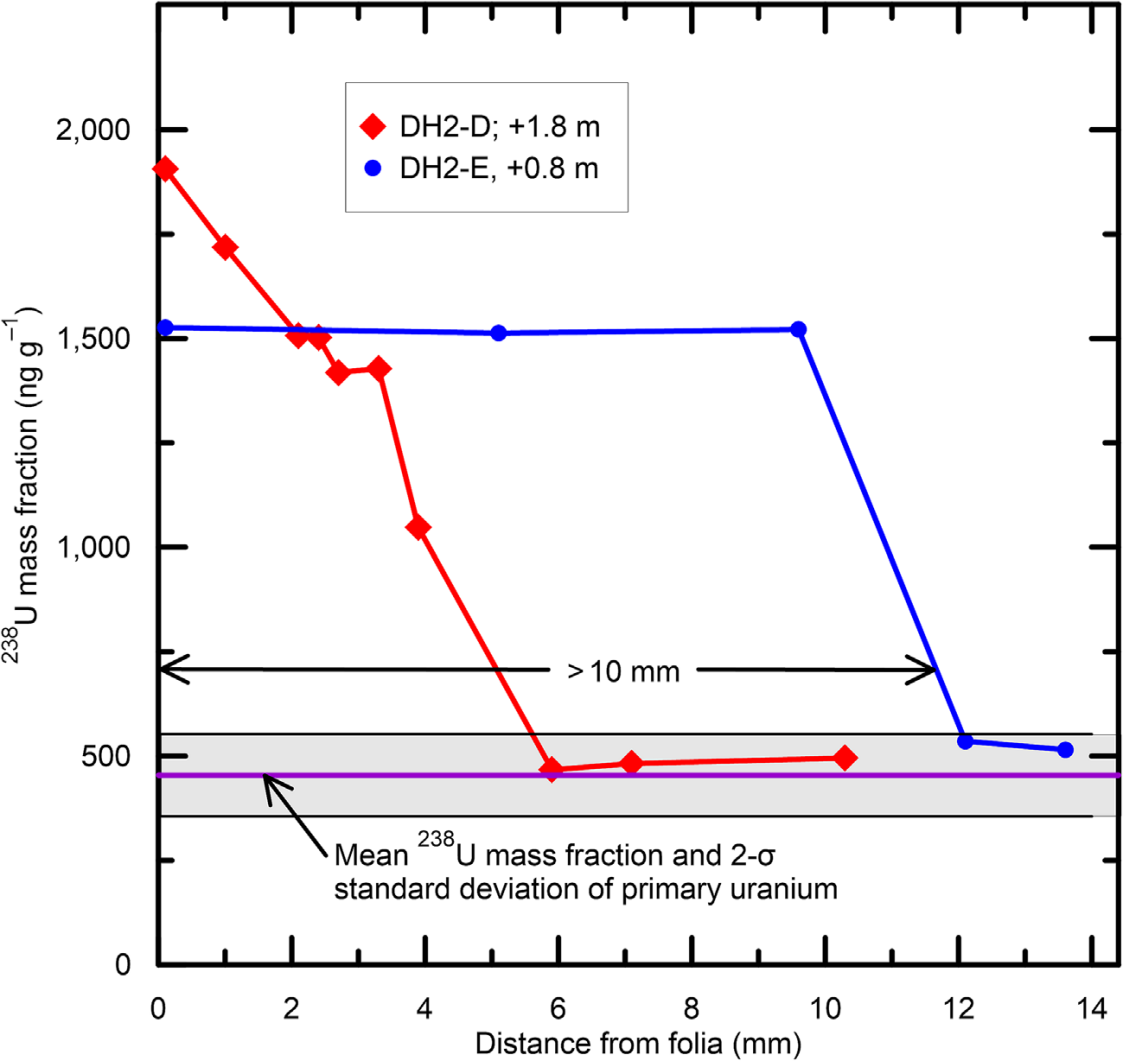
Crossplot of ^238^U mass fraction within 10 DH2‐D samples and 5 DH2‐E samples and their distance from adjacent folia near Termination I. The 10 DH2‐D samples range from DH2‐D1‐0 (^238^U mass fraction = 1906.5 ± 2.0 ng g^−1^) adjacent to folia layers (distance = 0 mm) at an elevation of +1.8 m above the modern water table to DH2‐D1‐5 (^238^U mass fraction = 495 ± 2 ng g^−1^) located 10.2 mm from the folia layers.^17^ The five DH2‐E samples range from DH2‐E‐0.4 (^238^U mass fraction = 1526 ± 2 ng g^−1^) adjacent other folia layers (distance = 0 mm) at an elevation of +0.8 m above the modern water table to DH2‐E‐13.9 (^238^U mass fraction = 515.0 ± 0.4 ng g^−1^) located 13.5 mm from the folia layers.^17^ [Color figure can be viewed at wileyonlinelibrary.com]

Secondary uranium can infiltrate at least 10 mm within subaerial cores (Figure 9). Within DH2‐E (+0.8 m), the mass fraction of ^238^U decreases from 1526 ± 2 ng g^−1^ in sample DH2‐E‐0.4 adjacent to folia to 1521 ± 2 ng g^−1^ in sample DH2‐E‐9.9. In accord with our postulated infiltration of secondary uranium, the concentration of uranium decreases nearly monotonically in cores DH2‐D and DH2‐E away from adjacent folia (Figure 9). At a sufficient distance from folia (6 mm for DH2‐D and 12 mm for DH2‐E in Figure 9), there is no evidence of secondary uranium enrichment, with ^238^U concentrations having decreased to that of primary uranium concentrations and with ^230^Th/U ages that are in general agreement with values in deep cores.

One might ask why mixed ^230^Th/U ages are found primarily in older calcite adjacent to a hiatus in precipitation and rarely in younger calcite adjacent to a hiatus. For example, all six DH2‐D samples in the 10 mm of calcite formed prior to folia deposition (61.4–80.5 mm in tab. S1 of Moseley et al.^17^) contain secondary uranium (Table S4), whereas in the 10 mm of calcite formed after the folia deposition, only one sample may contain secondary uranium (DH2‐D2‐1 at 80.7 mm).^17^ In DH2‐E, all five samples formed prior to folia deposition contain secondary uranium, whereas only one calcite sample formed after folia deposition contain secondary uranium (tab. S1 of Moseley et al.^17^ and Table S4). Near the end of interglacials, the water table rises, enabling calcite to precipitate over folia. Because there is no exposure to air with concomitant development of “punk” carbonate, a soft powdery material,^29^ there is no driving force for uranium to migrate through dense, freshly precipitated calcite. Thus, mixed ^230^Th/U ages seldom appear on the younger sides of calcite hiatuses.

Cores DH2‐D (+1.8 m) and DH2‐E (+0.8 m) differ in elevation by only 1 m, and one would expect coevally precipitated mammillary calcite (based on identical *δ* ^18^O values) to have the same ^230^Th/U age within analytical uncertainty. However, Figure 3 shows that coevally formed calcite samples from cores DH2‐D and DH2‐E (samples having the same *δ* ^18^O values) have ^230^Th/U ages that are in temporal disagreement by thousands of years in support of our hypothesis of secondary uranium being transported into calcite, resulting in mixed‐age samples.

Additional evidence of mixed‐age samples is that of samples that are out of stratigraphic order.^41^ For example, sample DH2‐D1‐M1 (8226 ± 22 a) in core DH2‐D (+1.8 m) is emphasized in red italic font in tab. S1 of Moseley et al.^17^ as a sample with “rejected ages as a result of drilling difficulties and being out of stratigraphic order.” DH2‐D1‐M1 (8226 ± 22 a) formed after samples DH2‐D1‐M2 (8026 ± 84 a) and DH2‐D1‐M3 (8012 ± 103 a) and should be younger than both of those samples but aged dated as older. Another sample out of stratigraphic order in tab. S1 of Moseley et al.^17^ is DH2‐D1‐22 having an age of 117 551 ± 371 a. This sample was formed after sample DH2‐D2‐1 having an age of 116 072 ± 415 a. The age of DH2‐D2‐1 should be older than that of DH2‐D1‐22, but it is younger. Five percent of the sample results published by Steidle et al.^21^ are identified as being out of stratigraphic order.

Our data evaluation identified five samples in core DH2‐D (+1.8 m) having ^238^U mass fractions between 511.3 and 564 ng g^−1^ and four samples in core DH2‐E (+0.8 m) having ^238^U mass fractions between 548 and 562 ng g^−1^. The ^238^U mass fractions of secondary uranium in these nine samples range from 29 to 86 ng g^−1^ (Table S4). For example, consider sample DH2‐D2‐7 (distance from top of core (dft) 101.4 in Wendt et al.^19^) having a ^230^Th/U age of 128 816 ± 421 a,^19^ a ^238^U mass fraction of 564.0 ± 0.6 ng g^−1^, and a *δ* ^18^O_VPDB_ value of −15.35‰.^16^, ^17^ The ^238^U mass fraction of primary uranium in coeval calcite is that of DH‐2 having a *δ* ^18^O_VPDB_ value of −15.35‰, and Figure S1 shows its age as approximately 136 700 a. Because Moseley et al.^17^ did not measure a DH‐2 (−21 m) sample having an age of 136 700 a, the mass fraction must be determined by linear interpolation of an older and a younger DH‐2 (−21 m) sample, namely, sample DH2‐8 having an age of 136 841 ± 442 a^17^ and a ^238^U mass fraction of 483 ± 0.4 ng g^−1^ and sample DH2‐8.1 having an age of 127 865 ± 398 a^17^ and a ^238^U mass fraction of 471 ± 0.4 ng g^−1^.^17^ The estimated ^238^U mass fraction of primary uranium formed at approximately 136 700 a is 483 ng g^−1^. Therefore, the ^238^U mass fraction of secondary uranium in sample DH2‐D2‐7 is about 564 ng g^−1^ − 483 ng g^−1^ = 81 ng g^−1^, which is shown in Table S4. For the 25 samples in Table S4 and 14 samples of Table S5 for which *δ* ^18^O_VPDB_ values have not been published, to be conservative, a ^238^U mass fraction of 556 ng g^−1^ is assumed. This value is the upper bound of the 2‐*σ* uncertainty of primary uranium (Figure 7).

The 25th and 75th percentiles and medians in Figure 5 of Devils Hole Cave 2 and Brown's Room calcite are all greater than those for deep calcite samples, strongly suggesting that these subaerial calcites also might all bear lesser amounts of secondary uranium contamination than found in Devils Hole Cave 2 folia samples. We evaluate 147 samples analyzed by Moseley et al.^17^ and Wendt et al.,^19^ having ages less than 296 ka, by using the sample stable isotope values to make a tie point to the ^238^U mass fraction of coeval samples from the DH‐2 record. We assess whether the Moseley et al.^17^ and Wendt et al.^19^ samples have excess (secondary uranium) above that of the coeval DH‐2 sample. Of 147 samples analyzed by Moseley et al.^17^ and Wendt et al.^19^ having ages less than 296 ka, our evaluation indicates that 46 have mixed ^230^Th/U ages (Table S4). We urge caution in using the mixed ^230^Th/U ages of these 46 samples and the assigned ^230^Th/U ages of *δ* ^18^O and *δ* ^13^C values. Table S5 lists 14 samples from Wendt et al.^19^ or Steidle et al.^21^ older than 296 ka having estimated secondary uranium mass fractions as high as 1115 ng g^−1^.

Schwarcz^41^ discusses the possibility of loss of uranium from samples and the result for uranium‐series ages. Table S6 tabulates 19 samples from Wendt et al.^19^ and Steidle et al.^21^ having very low ^238^U mass fractions, ranging between 43.7 ± 0.1 and 303.8 ± 0.3 ng g^−1^ and ages between 79 480 ± 280 and 823 178 ± 17 121 a. These anomalous mass fractions are less than the lower 3‐*σ* bound of mean primary uranium, which is 306 ng g^−1^. We suggest that weathering and transport of uranium by groundwater recharge and (or) condensation corrosion^29^ may explain these low uranium concentrations. We urge caution in using these published ages.

Early in our investigation, we thought that bat guano might be an important source of uranium, and the uranium concentrations of bat guano and soil were determined (Table S2). We now believe that bat guano has not been a significant contributor of uranium within Devils Hole vein calcite over the last half million or more years.

Future investigators may want to compare the concentration of uranium within calcite crystals and in grain boundaries. Deposition of secondary calcite or other uranium‐bearing minerals, either filling primary porosity or replacing calcite,^22^ may play a role. Uranium‐bearing fluids may have locally been driven by tidally fluctuating hydraulic heads, exploited minor fractures or other porosity in the mammillary calcite during low tides, and encountered crystallographically more favorable sites for adsorption and eventual mineral lattice incorporation.^46^, ^47^ Because the water seeping through the fractures and other porosity in the intertidal zone is at equilibrium with calcite, its mineralogical and geochemical fingerprint would be expected to be cryptic, possibly limited to the uranium enrichment.

### 4.2 Why might uranium be concentrated in folia?

Calcite and folia form under distinctly different hydrochemical and mineralogical conditions, the effect of which is often observable in the uranium chemistry of these geochronological materials. Calcite forms at least 1 m below the water table^5^, ^44^ from water that is slightly supersaturated with respect to calcite.^43^ In contrast, folia form at or above the water table in the intertidal zone of Devils Hole, which varies between 3 and 11 cm depending on the phase of the moon.^5^ The primary process responsible for folia precipitation is carbon dioxide outgassing at the folia surface as high partial pressure of CO_2_ in groundwater (log pCO_2_ = −1.9) equilibrates with atmospheric pressures (log pCO_2_ = −3.4).^5^, ^43^ Geochemical modeling using the Geochemist's Workbench,^48^ supplemented with thermodynamic data from Guillaumont et al.^49^ and Dong and Brooks,^50^ suggests that CaUO_2_(CO_3_)_3_^2−^ is the dominant uranium‐bearing aqueous species during folia precipitation throughout most of the outgassing process. Precipitation of calcite with trace amounts of U(VI), U^6+^ or U^+6^, during CO_2_ outgassing can be described by the generic reaction

0.98Ca^2+^ + 1.94HCO_3_^−^ + 0.01CaUO_2_(CO_3_)_3_^2−^ → Ca_0.99_(UO_2_)_0.01_CO_3_ + 0.97CO_2_ + 0.97H_2_O

As the partial pressure of CO_2_ decreases, the reaction progresses to the right, precipitating uranium‐bearing calcite. This reaction well describes the precipitation of uranium‐bearing calcite during CO_2_ outgassing, but it does not alone explain the higher concentrations of uranium found in folia. Conversely, the absorption of CO_2_ gas by condensed water films in the vadose zone drives this reaction to the left, causing the local dissolution of calcite—a process known as condensation corrosion^29^—and the remobilization of contained uranium. In fact, Dublyansky and Spötl^29^ concluded that condensation corrosion has been a localized but ubiquitous process in the speleogenesis of the Devils Hole system throughout its history. This fact requires careful examination of any assumptions about closed‐system behavior of calcite‐bound uranium in the subaerial zones at Devils Hole.

Uranium incorporation into calcite is a complex process involving initial adsorption of uranyl (UO_2_^2+^) as calcium uranyl triscarbonate species akin to aqueous Ca_2_UO_2_(CO_3_)_3_ followed by incorporation into the calcite lattice during calcite growth.^46^, ^47^ Calcite displays crystallographically preferred incorporation of uranyl (UO_2_^2+^) and other metals. Preferential incorporation of U(VI) occurs in growth steps on the ($10\bar{1}4$) surface plane of calcite—the most common growth surface and dominant cleavage. The magnitude of the differential uptake between preferred and non‐preferred sites is on the order of a factor of 2–6.^46^ The complexity of the adsorption/desorption of U(VI), its eventual incorporation into the calcite lattice, attendant structural changes, and kinetic limitations on the rates of these processes can influence the mobility of uranium in its early stages of incorporation in calcite.^46^ Published laboratory studies investigating the absorption of dissolved U(VI) on the surface of calcite and the rate of its incorporation into the calcite lattice found that the transition from adsorbed U(VI) to lattice‐bound U(VI) required around 200 h under conditions like those at Devils Hole.^51^

The implications of this crystallographically based preferential incorporation of U(VI) into calcite are profound. Calcite in speleothems typically grows in radially elongated grains with their *c*‐axis perpendicular to the growth surface.^52^ The calcite grains in the folia at Devils Hole are similarly oriented.^5^ In contrast, the *c*‐axis of the calcite in Devils Hole is roughly perpendicular to this orientation with the *c*‐axis at an angle greater than 60° to the direction of elongation.^5^ Thus, the orientation of the calcitic grains in folia and the greater porosity of folia compared with that of calcite are more conducive to preferential incorporation of U(VI) in folia as opposed to calcite. The ratio of the median uranium concentration of folia to that of deep calcite (Figure 5) is 4, which corresponds well to the range of differential uptake values for calcite determined in the laboratory of 2–6.^46^ Therefore, crystallographically based preferential uptake of U(VI) by calcite explains the enigmatic, anomalous concentrations of uranium found in folia at Devils Hole.

### 4.3 Corrected formation ages of subaerial calcite having ^230^Th/U ages <24 and 145–117 ka

Devils Hole (and Devils Hole Cave 2) is a natural laboratory, precipitating deep calcite continuously in oxygen isotopic equilibrium.^2^, ^3^ This well‐mixed system^22^ has no hiatuses due to meter‐wide fissures of extensional‐tectonic origin having very high fracture transmissivity,^22^, ^53^ alongside the supportive hydrogeologic setting.^22^, ^54^, ^55^, ^56^ Additionally, the temperature has remained constant to within ±1°C during the last half million years.^4^ As a result, widely separated coeval calcite samples have the same *δ* ^18^O (and *δ* ^13^C) values as exemplified by data between about 30 and 19 ka from widely separated cores DHC2‐3 (−25 m) and DHC2‐8 (−60 m) (fig. 4 of Coplen et al.^12^). The DH merged record combines the overlapping time series DHC2‐3, DHC2‐8, and DH‐11,^11^, ^12^ and this high‐accuracy DI‐IRMS, 563 000‐year chronology from continuously submerged calcite is termed the Devils Hole “gold‐standard” *δ* ^18^O time series.

The formation ages of 13 DH2‐D (+1.8 m) and 8 DH2‐E (+0.8 m) calcite samples with published *δ* ^18^O and *δ* ^13^C values^16^ and ^230^Th/U ages between 145 and 117 ka or between 24 and 4.9 ka were corrected using the *δ* ^18^O time series of deep core DH‐2 (−21 m)^16^ or of deep core DHC2‐8 (−60 m) (tab. 6 of Coplen et al.^12^), and they are shown in Table 1. The deep cores represent a continuously precipitated chronology because they formed under static, continuously submerged conditions throughout the time period that they record. Therefore, they should represent the “gold‐standard” *δ* ^18^O time series against which other proposed time series need to be compared and/or reconciled. In contrast, the subaerial folia, flowstone, and exposed calcite have all spent substantial parts of their history in a dynamic environment with wetting and drying cycling and obvious mobility of uranium as shown below. Consider sample DH2‐E‐9.9 from core DH2‐E (+0.8 m) that has a *δ* ^18^O_VPDB_ value of −16.06‰,^16^ a *δ* ^13^C_VPDB_ value of −2.02‰,^16^ and a ^230^Th/U age of 7168 ± 20 a.^17^ To determine the corrected age, one determines an age from the DHC2‐8 *δ* ^18^O_VPDB_ time series because the *δ* ^18^O_VPDB_ time series is more sensitive than the *δ* ^13^C_VPDB_ time series. For a *δ* ^18^O_VPDB_ value of −16.06‰, sample DHC2‐8‐9771 in tab. 6 of Coplen et al.^12^ has a *δ* ^18^O_VPDB_ value of −16.15‰ and an age of 18.3 ka. Sample DHC2‐8‐9770 in tab. 6 of Coplen et al.^12^ has a *δ* ^18^O_VPDB_ value of −15.86‰ and an age of 17.8 ka. The interpolated age is 18.1 ka, which is listed in the fifth column of Table 1. The ages of these 21 samples in Table 1 are too young by between 1500 and 12 000 years, which is far outside the 2‐*σ* measurement uncertainties published by Moseley et al.^17^ of 22 to 508 years.

**TABLE 1:** Formation ages of selected DH2‐D and DH2‐E samples based on a comparison of their *δ* ^18^O and *δ* ^13^C values with those of DHC2‐8 or DH‐2.

(1)	(2)	(3)	(4)	(5)	(6)
Sample ID	^230^Th/U age (a)	*δ* ^18^O_VPDB_ (‰)	*δ* ^13^C_VPDB_ (‰)	Age estimated from coeval DHC2‐8 or DH‐2 *δ* ^13^C and *δ* ^18^O time series (ka)	Difference in ages between columns 5 and 2 (years)
DH2‐D1‐0	4896 ± 45	−15.72	−2.19	16.9	12 000
DH2‐D1‐1	7945 ± 75	−16.00	−2.35	18.0	10 000
DH2‐D1‐M1	8226 ± 22	−16.10^a^	−2.34^a^	18.2	10 000
DH2‐D1‐M2	8026 ± 84	−16.11	−2.33	18.2	10 200
DH2‐D1‐M3	8012 ± 103	−16.05	−2.33	18.2	10 200
DH2‐D1‐M4	8808 ± 38	−16.03	−2.25	18.2	9400
DH2‐D1‐M5	12 929 ± 95	−16.50	−2.12	23.3	10 400
DH2‐D2‐6	121 404 ± 411	−14.68	−2.78	125.5	4100
DH2‐D2‐6.1	122 861 ± 304	−14.87	−3.01	128.5	5600
DH2‐D2‐7	128 751 ± 421	−15.35	−2.88	136.7	7900
DH2‐D2‐7.2	134 756 ± 418	−16.25	−2.31	143.0	8200
DH2‐D2‐8	138 468 ± 476	−16.44	−2.40	144.6	6100
DH2‐D2‐8.1	139 739 ± 443	−16.55	−2.41	146.0	6300
DH2‐E‐0.4	5723 ± 21	−15.84	−1.96	17.7	12 000
DH2‐E‐5.4	6149 ± 16	−16.05	−2.21	18.1	12 000
DH2‐E‐9.9	7168 ± 20	−16.06	−2.02	18.1	10 900
DH2‐E‐91.9	117 549 ± 508	−15.30	−2.72	119.0	1500
DH2‐E‐96.9	121 642 ± 349	−14.70	−3.03	124.0	2400
DH2‐E‐98.9	129 677 ± 449	−14.78	−3.03	132.5	2800
DH2‐E‐99.9	132 930 ± 392	−15.56	−2.62	137.7	4800
DH2‐E‐101.9	136 894 ± 422	−16.11	−2.48	142.1	5200

*Note*: Column 2 data from Moseley et al.^17^ and Wendt et al.^19^ Column 3 and 4 data from Moseley et al.^16^ Column 5 determined using *δ* ^18^O values of DHC2‐8^12^ and *δ* ^18^O values of Moseley et al.^16^ or *δ* ^13^C and *δ* ^18^O values of DH‐2.^16^

^a^ Extrapolated value for sample out of stratigraphic order.

The *δ* ^18^O_VPDB_ values of DHC2‐8 samples are nearly constant during the 12 500‐year interval between 17 and 4.5 ka, ranging between −15.90‰ and −15.75‰ (Figure 3). However, this plateau of nearly constant *δ* ^18^O values appears to be missing from the *δ* ^18^O_VPDB_ time series of DH2‐D (+1.8 m) and DH2‐E (+0.8 m) (Figure 3 and Figure S1) as noted by Coplen.^57^ We note that the *δ* ^18^O_VPDB_ values of the youngest surfaces of DH2‐D (+1.8 m) and DH2‐E (+0.8 m) are as much as 0.6‰ too positive (Figures 3 and 4A,B). This may be the result of condensation corrosion or exposure of calcite to air as the water table lowers with concomitant development of “punk” carbonate, a soft powdery material.^29^

### 4.4 Duration of the last interglacial *δ* ^18^O peak

Moseley et al.^16^ determined the duration of the last interglacial in the DH2‐D *δ* ^18^O time series to be 6000 years (127–121 ka), considering just the portion of the peak that records the most positive *δ* ^18^O values. This value is about half that determined by Winograd et al.^7^, ^9^, ^13^ from deep cores. The overly small duration of the width of this *δ* ^18^O‐time‐series peak in subaerial core DH2‐D (+1.8 m) can be explained by the fact that precipitation of calcite ceased at about 125.5 ka with sample DH2‐D2‐6 (Table 1) as the water table descended in response to the onset of interglacial climate and not at 121 ka. Correction of the DH2‐D mixed ^230^Th/U age when calcite precipitation ceased approximately doubles the 6000‐year duration of Moseley et al.^16^ to reconcile it with that of deep cores^7^, ^9^, ^13^ as shown in Figure S3.

One can also define the duration of the last interglacial as the time between the Termination II midpoint (defined by *δ* ^18^O_VPDB_ = −15.7‰) and the glacial inception midpoint (defined by *δ* ^18^O_VPDB_ = −15.4‰).^16^ One can use measurements of DH‐2 of Moseley et al.^16^, ^17^ to determine that the duration of the last interglacial is (139.8 ± 0.9 ka) − (118.0 ± 0.4 ka) = 21 800 years (tab. S2 of Moseley et al.^17^). This value is in good accord with the value of 21 800 years determined from DH‐11.^12^ Thus, the full duration of the last interglacial as recorded by the *δ* ^18^O time series of DH2‐D, measured by Moseley et al.,^16^ and that of DH‐11, measured by Ludwig et al.^6^ and Winograd et al.,^7^, ^9^ are reconciled.

### 4.5 Paleoclimate relevance

The paleoclimatic relevance of the half‐million‐year‐long *δ* ^18^O time series from Devils Hole in the southern Great Basin of North America has been debated for decades. On one hand, this time series is thought to be compatible with orbital forcing of the ice ages.^16^, ^18^, ^20^, ^58^, ^59^, ^60^ On the other hand, this time series is stated to be a regional southern Great Basin record.^1^, ^7^, ^13^, ^61^

Adjustment of the mixed ^230^Th/U ages of DH2‐D (+1.8 m) and DH2‐E (+0.8 m) samples^17^, ^19^ leads to reconciliation of these ages with the 1988–2011 Devils Hole *δ* ^18^O chronology (Table 1). The adjusted ages support the contention by Winograd et al.^7^ that the middle of the major shift to more positive *δ* ^18^O values in the Devils Hole paleoclimate record—139.8 ± 0.9 ka (2‐*σ* uncertainty), as published in tab. S2 of Moseley et al.^17^ for samples from core DH‐2—is a proxy of a major sea‐surface‐temperature (SST) warming off California,^1^, ^61^ an event that preceded glacial Termination II by approximately 10 000 years. We consider this measurement of 139.8 ± 0.9 ka^17^ to be the preferred measurement of this *δ* ^18^O midpoint. The middle of the *δ* ^18^O increase during the last glacial to interglacial transition (−16.2‰, Figure 3) precedes Termination I by about 5000 years.^7^ Based on high‐accuracy *δ* ^18^O measurements of subaerial calcite having mixed ^230^Th/U ages and of continuously submerged calcite, we rule out any possibility that the gold‐standard *δ* ^18^O time series is consistent with orbital forcing. Rather, we conclude conclusively that the Devils Hole *δ* ^18^O time series is a regional record as stated by Herbert et al.^61^ and Winograd et al.^7^

### 4.6 Great Basin composite dripstone record (Leviathan chronology)

The southern Great Basin composite dripstone record (Leviathan chronology),^58^, ^59^, ^60^ developed from speleothems from three Nevada caves (Lehman Caves, Leviathan Cave, and Pinnacle Cave) shown in Figure 1, ought to be in sync with the Devils Hole record because they have the same general source of recharge.^16^, ^54^ Because the Devils Hole *δ* ^18^O time series is a regional record (see Section 4.5), the Leviathan chronology must also be a regional southern Great Basin record. However, the Leviathan chronology supports an age of 132 ka for Termination II that is too young by about 8100 years (Figure S1). The duration of the last interglacial *δ* ^18^O peak of the southern Great Basin composite dripstone record (about 11 000 years) is not in agreement with that from cores DH‐2 and DH‐11 of 21 800 years^6^, ^7^, ^9^ (see Section 4.3). We conclude that the temporal disagreement between the southern Great Basin composite dripstone record and the Devils Hole chronology from deep calcite can be explained by post‐formation uranium addition. We suggest that uranium transport may be an underappreciated geochemical mechanism, especially for speleothems and subaerial carbonates having hiatuses.

### 4.7 Water‐table hydrographs and paleo‐groundwater recharge

In 1994, a 120 000‐year time series of water‐table height at Devils Hole from alpha spectrometric ^230^Th/U measurements of folia, flowstone, and calcite at Brown's Room was published.^15^ We have substantial concerns about the accuracy of the ages of Brown's Room samples because of the following: (1) Most of the measurements are on folia, and these folia have a median ^238^U mass fraction of 1112 ng g^−1^ (*n* = 23) with values as high as 2890 ng g^−1^ (Figure 5), suggesting substantial transport of secondary uranium; (2) Moseley et al.^17^ concluded that folia “cannot be dated using our current methods (Table S1)” after measuring widely discordant ages of 145 633 ± 533, 270 614 ± 1823, 117 559 ± 412, and 129 522 ± 422 a (the peak‐to‐peak variation is 153 ka) on four DH2‐D (+1.8 m) adjacent folia samples that should all have had ages of about 125 ka, based on the time of cessation of formation of calcite (Table 1); (3) flowstone samples^5^, ^15^, ^45^ are porous to very porous, and their median ^238^U mass fraction is 1535 ng g^−1^ (*n* = 6), which is similar to the median of Devils Hole Cave 2 folia (Figure 5), strongly suggesting transport of secondary uranium; (4) calcite samples show evidence of secondary uranium (Figure 5), and a substantial fraction of these samples may be too young because their median ^238^U mass fraction is 612 ng g^−1^ (*n* = 9), which is greater than that of high‐^238^U calcites (i.e., 606 ng g^−1^, which is 3*σ* above the mean value of primary ^238^U of 456 ng g^−1^ as shown in Figure 7); and (5) 10% of the published ages of Szabo et al.^15^ are out of stratigraphic order, which is evidence of mixed‐age samples.^41^

We would be remiss if we did not try to estimate the ages of Brown's Room samples. Therefore, in a manner identical to that used to estimate ages of samples from cores DH2‐D and DH2‐E (Table 1), the *δ* ^18^O and *δ* ^13^C values of nine Brown's Room samples, collected between 9 and 0 m above the modern water table (Table S7), were used to estimate their formation ages, assuming that calcitic folia and flowstone and calcite samples precipitated in oxygen isotopic equilibrium at the same temperature that DHC2‐8 (−60 m) formed. This is a reasonable assumption because Brown's Room is an enclosed void, and its relative humidity is near 100%.^44^ Estimated formation ages ranged from 29.8 to 18.1 ka (Table S7). These data suggest that the ^230^Th/U ages of Szabo et al.^15^ are too young by between 3500 and 11 600 years (Table S7).

A comparison of the ages and sample height above the modern water table of Brown's Room samples between 30 and 0 ka, estimated via their *δ* ^18^O and *δ* ^13^C values (Table S7) and published by Szabo et al.,^15^ is shown in Figure S4A. The major difference between these time series is that the water level decreased from 9 to 0 m between 19.5 and 1.2 ka according to Szabo et al.^15^ and between about 24.5 and 18 ka (Table S7 and Figure S4A) according to our evaluation. According to Szabo et al.,^15^ the water level declined several meters during the Holocene. According to our findings (Table S7 and Figure S4A), the water level had declined to its current level by about 18 ka, during the Pleistocene and well before the start of the Holocene. Why the decline in water table from 4 to 0 m occurred so quickly at about 18 ka (Figure S4A) is unknown. However, a decrease in moisture availability beginning at approximately 18 ka is in accord with the abrupt beginning of increasing *δ* ^13^C values in the DHC2‐8 time series at 18 ka (Figure S4B) because increasing *δ* ^13^C values have been proposed to reflect a period of decreasing density of vegetation.^27^

To investigate moisture availability in the southwest United States, Wendt et al.^18^, ^62^ reconstructed past water‐table elevations of Devils Hole by dating thin calcite layers. As shown above, many of the 145–115 ka ^230^Th/U ages used by Wendt et al.^18^, ^62^ to construct their paleowater‐table hydrograph are too young (Table S4). Our evaluations suggest that subaerial calcite was last precipitated in Devils Hole Cave 2 about 18 ka (Table 1) and folia were last precipitated in Brown's Room about 18 ka (Table S7 and Figure S4A). We conclude that there are no data from Devils Hole for a paleowater‐table hydrograph within the last approximately 17–18 kiloyears. We conclude that many of the ages used by Wendt et al.^18^, ^62^ are mixed ages that are as much as 12 000 years too young because of post‐depositional enrichment of uranium (Tables S4 and S7). Wendt et al.^62^ extended the Wendt et al.^18^, ^19^ paleowater‐table hydrograph to 475 ka using at least 13 samples that we suggest are mixed‐age samples containing secondary uranium with mass fractions of uranium greater than 606 ng g^−1^ (fig. 2B of Wendt et al.^62^). As shown in figs. 2 and 3 of Wendt et al.,^18^ the post‐20‐ka hydrograph of Wendt et al.^18^ is in good agreement with the hydrograph from Brown's Room.^15^ We suggest that both are incorrect.

Jackson et al.^63^ present a 350 000‐year history of groundwater recharge in the southern Great Basin. This history relies on the water‐table records of Szabo et al.^15^ and Wendt et al.,^18^, ^19^ which are mixed‐age samples. At about 15 ka, Jackson et al.^63^ show a water‐table elevation in Devils Hole of about 5 m with a total simulated recharge of about 5.3 × 10^7^ m^3^ year^−1^. Our Brown's Room findings suggest that the water table had descended to 0 m by approximately 18 ka. At 15 ka, we would estimate a total simulated recharge of approximately 2.6 × 10^7^ m^3^ year^−1^ for a water‐table level of 0 m according to fig. 2b of Jackson et al.,^63^ which is about half that shown at 15 ka by Jackson et al.^63^ We would expect a similar concerning scenario during the interglacial between approximately 140 and 120 ka because the ages of DH2‐D samples are as much as 8200 years too young during this interval according to our evaluations (Table 1).

Lowenstein et al.^64^ published a paleohydrologic history of the southern Great Basin. Numerous water‐table elevations in this publication are based on calcite samples from Devils Hole Cave 2 having mixed ^230^Th/U ages with secondary uranium sourced. These include samples from core H (+9.5 m), core I (+8 m), core J (+5.3 m), core F (+3.1 m), core D (+1.8 m), core E (+0.8 m), and core O (0 m). For example, Lowenstein et al.^64^ state that the Devils Hole water table dropped to the modern water level by 120.36 ± 0.45 ka. This date is based on sample dft = 139 mm of core O having a ^238^U mass fraction of 18 923 ± 3.3 ng g^−1^ (tab. S1 of Wendt et al.^19^). This is a ^230^Th/U mixed‐age sample having an estimated secondary uranium mass fraction of 18 367 ng g^−1^ (Table S4). We suggest that the secondary uranium is sourced from adjacent folia 1 mm away (dft = 138 mm) (tab. S1 of Wendt et al.^19^). Their stated +9.5‐m elevation at 144.32 ± 0.58 ka is based on calcite sample dft = 19 mm of core H having a ^238^U mass fraction of 818.2 ± 1.3 ng g^−1^ (tab. S1 of Wendt et al.^19^). This is a ^230^Th/U mixed‐age sample that has an estimated secondary uranium mass fraction of 262 ng g^−1^ (Table S4). We suggest that the secondary uranium is sourced from adjacent folia (dft = 18 mm) (tab. S1 of Wendt et al.^19^). Lowenstein et al.^64^ state that the Devils Hole water table rose 5.3 m above its present level by 201.8 ± 0.9 ka and remained above that level until 136.3 ± 1.4 ka. Their date of 136.3 ± 1.4 ka is based on sample dft = 189 mm of core J with a ^238^U mass fraction of 650.3 ± 1.6 ng g^−1^ (tab. S1 of Wendt et al.^19^). This is a ^230^Th/U mixed‐age sample that is too young having an estimated secondary uranium mass fraction of 94 ng g^−1^ (Table S4). The water‐table elevation record of Devils Hole in fig. 2A of Lowenstein et al.^64^ appears to be based on several mixed ^230^Th ages between 15 000 and 0 a, for example, from core F (+3.1 m), core E (+0.8 m), core D (+1.8 m), and core O (0 m).^19^ Concentrations of secondary uranium in these core samples are estimated in Table S4, and they are as high as 37 046 ng g^−1^, substantially higher than the value of 456 ± 100 ng g^−1^ for primary uranium.

Steidle et al.^20^, ^21^ present a moisture availability and groundwater recharge record over the last 750 ka, which is based on uranium‐series ages. This record is suspect because many of the ages are mixed uranium‐series ages having secondary uranium. Assuming a mass fraction of 556 ng g^−1^ for primary uranium within calcite, Table S5 lists 12 samples from Steidle et al.^21^ having estimated secondary uranium mass fractions as high as 1115 ng g^−1^.

### 4.8 Evidence for cessation of precipitation of calcite in Devils Hole Cave 2

In 1992, it was recognized that precipitation of calcite collected from Devils Hole stopped at about 50–60 ka even though the water in this cave remained supersaturated with respect to calcite.^9^ Riggs^65^ proposed that Devils Hole opened to the surface at about 60 ka with the introduction of inorganic ions from the land‐surface environment or organic compounds generated by the aquatic flora and fauna that colonized the newly available habitat, terminating calcite precipitation from slightly supersaturated groundwater. To find calcite younger than 50 ka, Winograd et al.^7^ moved up the hydraulic gradient to Devils Hole Cave 2. There, divers discovered younger calcite (DHC2‐3) with an age of about 20 ka by diving to a depth of 25 m and moving up the hydraulic gradient about 70 m from the water‐table entrance of Devils Hole Cave 2 (Figure 2). To obtain even younger calcite (DHC2‐8; 30.4–4.8 ka), divers needed to move up the hydraulic gradient 110 m and dive to a 60‐m depth (Figure 2). Figure S5 shows the estimated minimum zone of cessation of precipitation of calcite since approximately 18–20 ka based on reevaluation of the age of the outer surface of DHC2‐3.^11^ As proposed for Devils Hole by Riggs,^65^ we suggest that Devils Hole Cave 2 opened to the surface at about 18–20 ka, prompting introduction of inorganic ions from the land‐surface environment or organic compounds generated by the aquatic flora and fauna that colonized the newly available habitat, thus preventing calcite precipitation from slightly supersaturated groundwater. If this suggestion is correct, calcite collected at or above the modern water table (Figure 2 and Figure S5) should have no ages younger than approximately 18 ka, which is in reasonable accord with the estimated ages of the youngest surfaces of subaerial cores DH2‐D (+1.8 m) and DH2‐E (+0.8 m) (Table 1). We know of no sample collected from the zone of cessation of precipitation of calcite in Table S4 having a demonstrable non‐mixed ^230^Th/U age less than about 18 ka (Table 1) as estimated by *δ* ^18^O values or 20.62 ± 0.49 ka as measured by thermal ionization mass spectrometry (TIMS).^7^

### 4.9 Using *δ* ^18^O measurements to identify speleothems having mixed ^230^Th/U ages

Based on the findings herein, it is suggested that comparing the *δ* ^18^O time series of closely spaced speleothems can be a good method to identify speleothems having mixed ^230^Th/U ages. If *δ* ^18^O time series differ by more than the analytical error, one should investigate for migration of secondary uranium with resulting mixed ^230^Th/U ages. The coeval sample, based on *δ* ^18^O values, having the younger age may be the mixed‐age sample because of input of younger uranium.

## 5 CONCLUSIONS

DI‐IRMS *δ* ^18^O measurements of dense, continuously precipitated, and submerged ^230^Th/U‐dated vein (mammillary) calcite over 563 000 years at Devils Hole, Nevada, created an unparalleled “gold‐standard” *δ* ^18^O time series. This time series, against which other proposed time series need to be compared and/or reconciled, has been used as a calibrator to correct mixed ^230^Th/U ages in vein calcite from two cores cyclically exposed by water‐table decline during glacial–interglacial transitions. These exposed calcite samples may contain excess uranium sourced from adjacent folia—porous carbonate deposits that form within the 3–11‐cm intertidal range of the water table and can have ^238^U mass fractions as high as 4800 ng g^−1^. Secondary uranium mobilized from the formation or dissolution of these folia has migrated up to 10 mm into the vein calcite from the folia–calcite boundary, resulting in mixed‐age samples that can be up to 12 000 years too young.

Out of 147 high‐precision ^230^Th/U age measurements conducted on calcite samples from nine cores at or above the modern water table (younger than 296 ka), our analysis shows that 46 samples contain secondary uranium. The presence of secondary uranium led to mixed ^230^Th/U ages that are up to 12 000 years too young. As a result, several studies that previously concluded that the Devils Hole *δ* ^18^O chronology aligns with orbital forcing are not supported by these corrected data.

Reconciliation of the *δ* ^18^O time series from core DH2‐D (+1.8 m) with the continuously submerged calcite record suggests that the timing and duration of glacial terminations in the Devils Hole *δ* ^18^O series do not correspond with orbital forcing. Instead, we conclude that the Devils Hole *δ* ^18^O time series reflects a regional climate record in the southern Great Basin of North America, indicating SST variations off California. These variations preceded the last and penultimate deglaciations by 5000 years to approximately 10 000 years. Consequently, the Leviathan chronology, another southern Great Basin *δ* ^18^O record, which was previously thought to align with orbital forcing, is significantly divergent from the Devils Hole record. The middle of the *δ* ^18^O increase before Termination II in the Leviathan chronology is approximately 8100 years too young. This discrepancy suggests that the Leviathan chronology is also a regional paleoclimate record affected by unrecognized post‐formation uranium addition, which has made the timeline appear younger than it is.

We find no evidence of vein calcite precipitation in Devils Hole Cave 2 within the past 17 000–18 000 years. Thus, published studies on the Devils Hole water‐table hydrographs, paleo‐moisture availability, and groundwater recharge, which are based on calcite with mixed ^230^Th/U ages, are as much as 12 000 years too young.

Several high‐profile publications did not recognize and correct for post‐formation uranium addition in Devils Hole cores collected at or above the modern water table, as well as in speleothems from Lehman Caves, Leviathan Cave, and Pinnacle Cave in Nevada. Therefore, we conclude that post‐formation uranium addition is a significant and unrecognized issue as a contributing source of variance in the uranium‐series dating of subaerial cores and speleothems. We suggest that comparing *δ* ^18^O time series from closely spaced speleothems can be an effective method to identify samples with mixed ^230^Th/U ages.

## AUTHOR CONTRIBUTIONS

**Tyler B. Coplen**: Conceptualization; Investigation; Writing—original draft; Formal analysis; Methodology; Validation; Data curation. **Robert R. Seal:** Formal analysis; Validation; Writing—original draft; Writing—review & editing; Methodology; Investigation. **Lauren T. Reid:** Writing—review & editing; Data curation. **James A. Jordan:** Methodology; Writing—review & editing; Investigation. **Adam C. Mumford:** Methodology; Writing—review & editing; Investigation.

## Data Availability

Data for this study are available.^14^
